# The Impact of Counseling on the Dignity of Older People: Protocol for a Mixed Methods Study

**DOI:** 10.2196/45557

**Published:** 2023-06-23

**Authors:** Ho Yeow Hung, Azlinda Azman, Paramjit Singh Jamir Singh

**Affiliations:** 1 School of Social Sciences Universiti Sains Malaysia Penang Malaysia

**Keywords:** counseling, dignity, elderly, emotional management, psychological, Singapore

## Abstract

**Background:**

Psychological counseling is perceived as a treatment that could significantly improve older individuals’ psychological and behavioral functioning. There is a dearth of information on the impact of psychological counseling on preserving dignity and facilitating good aging among older people in Singapore.

**Objective:**

The objectives of this study are as follows: (1) to assess advance care planning among older people and their perception of life and health, end of life, and end-of-life care; (2) to explore older people’s accessibility and receptiveness toward counseling; (3) elucidate older people’s perspectives on counseling and its impact on emotional management and decision-making; (4) to assess older people’s competencies in emotional management; and (5) to propose an intervention model for enhancing older people’s well-being and dignity through psychological counseling in Singapore.

**Methods:**

A mixed method study design involving quantitative and qualitative methods will be used. Older individuals receiving some form of preventive, primary, or long-term care in the community through voluntary welfare organizations from the senior activity centers located in eastern Singapore participated in the qualitative phase. Six older individuals from each senior activity center have been enrolled for the interview phase to explore 6 components: the Advanced Care Planning (ACP) booklet, Trait Meta Mood Scale (TMMS), accessibility and receptiveness toward counseling, and emotional management and decision-making. The ACP, an instrument designed to assess advanced care planning among older individuals, and the TMMS, an instrument developed to measure meta-mood experience and emotional management, were used in the quantitative phase among 100 participants. The data will be analyzed thematically using NVivo version 12, whereas descriptive statistics and a 2-tailed, 1-sample *t* test will be conducted in SPSS (version 25; IBM Corp) for empirical data analyses.

**Results:**

The qualitative phase, which involves a semistructured interview, has been completed among 20 older individuals aged 66-86 years. Thematic analysis of the data is still ongoing. Meanwhile, the quantitative phase commenced on March 22, 2022, with 100 participants providing signed informed consent to participate in the study. The study is expected to be completed by March 2023.

**Conclusions:**

The mixed methods study will document the current awareness of ACP, accessibility and receptiveness toward counseling, and the potential use of psychological counseling in enhancing well-being and dignity among older people in Singapore. The research findings will benefit policy makers in their decision-making when attempting to mitigate the potential barriers to seeking counseling assistance among older people.

**International Registered Report Identifier (IRRID):**

DERR1-10.2196/45557

## Introduction

### Overview

The current rising older population in Singapore reflects that one-fourth and half of the population will be older than 65 years by 2030 and 2050, respectively [1]. Previous studies have demonstrated that older people experience diverse aging-related complexities that require counseling intervention: depression, anxiety, grieving, loss of functional health abilities, financial dependence following retirement, social isolation, intergenerational and marital conflicts, and mental and emotional challenges [2,3]. These issues have been increasingly reported among older parents filing for financial maintenance with the tribunal court [4]. Hence, the prevalence of depressed older adults is expected to rise from 19,000 to 50,000 by 2020 [3].

Depression among older people is one of the main causes of suicide in comparison to other age groups globally. Despite implementing relevant policies and high awareness of mental health issues in Singapore to reduce recent suicide rates, the numbers among older adults remain relatively high [5]. For instance, individuals aged 60 years and older accounted for 25%-30% of suicide cases reported in Singapore in 2016, rising to 35.7% in 2017 [5]. Chan [6] also reported that older individuals with extensive depressive symptoms are among the main focus of the Singaporean government.

Recent studies have disclosed the need to accommodate the impacts of demographic shifts on Singapore’s health care eldercare initiatives [7]. The Singapore Ministry of Health provides intermediate and long-term services to address these concerns about the country’s aging population. These policies come in the form of community and residential health care services. While community clinics include hospitals for the chronically ill and nursing homes that are responsible for the provision of residential health care services, center-based health care services are provided by rehabilitation, day care, and psychiatric centers [8].

The Singaporean government has taken diverse approaches to provide better care for older people. For instance, the government is on the verge of increasing the number of nursing home beds to 14,000, accounting for a 50% increase from the report in 2010 [9]. These measures were based on the expected increase in demand for beds as the aging population continues a rising trend [6]. The Ministry of Health has also used smart technologies to facilitate cost-effective eldercare services [10]. Kong and Woods [10] recommended collaborative and contextually sensitive strategies to design and develop smart solutions for older people. Likewise, Ansah et al [11] suggested that resource planners and policy makers proactively review health needs and improve the dignity of the aging population in Singapore.

Despite the availability of previous treatment plans for psychiatric care, counseling is a current treatment approach, especially in recent cases of depression among older people [12]. Psychological counseling is presently perceived as a treatment that could significantly optimize individuals’ psychological and behavioral functioning [12,13]. As most older people spend the remainder of their lives within the community, insights into how residents uphold their sense of dignity within the community are valuable to managing aging stressors. An aging population creates both opportunities and pressures for Singapore’s welfare and health sectors. More importantly, the risk of dignity loss is high among older people during acute hospital care [14].

Dignity is vital to older individuals and their caregivers. However, it remains unclear how older people define dignity or dignified care and how it is influenced by psychological counseling [15]. Past research investigated how older people’s dignity could be safeguarded or enhanced by facilitating individual autonomy [16], providing personalized care, reinstating control, portraying respect, and conducting advocacy and sensitive listening [13]. Identifying older people as individuals and the aspects and values important to them are also fundamentals of care that could potentially conserve their dignity before death [13].

### Why Is It Important to Conduct This Study?

Approximately 30% of the 400 suicide cases reported in Singapore in 2019 involved individuals aged 60 years or older [7]. The number of Singaporeans older than 65 years who lived alone increased from 47,000 (9.7%) in 2016 to 67,600 (11.6%) in 2019 [7]. Previous studies also revealed that older adults with ailments and disabilities are exposed to cumulative suicide risks [17] and depression symptoms [3]. As the implications of aging reflect mental health complexities, aging is affected by several losses that require constant adjustments. Aging-oriented losses involve potential stress factors, specifically for older adults with compromised health conditions. Past studies have also highlighted how ailments and disabilities pose risks to older people and how mitigating factors could substantially reduce diseases and mortality rates among older adults [18].

Given the rising aging population, Singapore’s government health care is unable to accommodate all older people in hospitals and nursing homes. Hence, the Ministry for Social and Family Development developed a policy for older adults to “age in place,” in which they could independently live in their homes and communities. The policy encourages good health by increasing preventive, primary, and long-term care [19]. Additionally, these various types of care offer health and social care needs to support the families of older adults and can be successfully implemented with effective psychological counseling. As physical and cognitive activities represent essential and modifiable protective factors for cognitive decline and dementia, counseling interventions could assist in preventing these issues in older people [3,11].

There is a dearth of information on the effectiveness of counseling older adults in the Singaporean context. However, certain issues have been reported in the literature to impact accessibility and receptivity toward counseling services among the aging population. For instance, the cultural stigmatization associated with psychological counseling services specifically among older people [20,21] makes individuals believe seeking professional counseling is socially unacceptable [22].

Thus, older adults are less inclined and receptive to counselling, even when such assistance is necessary, based on their low awareness of the benefits. Likewise, older adults tend to be deterred by health and disability issues during counseling sessions due to accessibility and receptiveness. These concerns could adversely affect older individuals’ daily functioning and dignity preservation levels.

Counseling could be advantageous for older adults with emotional and psychological issues as a form of antidepressant and for ameliorating various mental disorders for behavioral shifts [23]. Counseling can also assist older adults’ emotional management and decision-making processes by addressing psychological issues. Hence, counseling in emotional management and decision-making processes would enhance the individuals’ sense of dignity in life and aging-oriented transitions. Apart from establishing government-funded counseling services under the family service centers [20], Singapore has also developed social welfare agencies, religious institutions, educational schools, and other governmental institutions that offer counseling services to the general public. Counseling services are also provided by multiple social service agencies and public and private hospitals [21].

Nevertheless, these various counseling services have not been elucidated in the context of Singapore as the impact of psychological counseling in preserving dignity and facilitating good aging among older people remains unknown. In other words, the experience and benefits of the counseling services provided to older citizens are underreported. To fill this research gap, this study aims to propose an intervention model for enhancing older people’s well-being through counseling.

### Research Objectives

The objectives of this study are as follows: (1) to explore older people’s accessibility and receptiveness toward counselling; (2) to assess the improvement of older people’s dignity in emotional management and decision-making processes following psychological counseling; and (3) to propose an intervention model for enhancing older people’s well-being through psychological counseling.

### Research Questions

Due to the paucity of data regarding the impact of counseling services on older adults’ dignity and the aging process, this study aims to answer the following questions:

- Do older people in Singapore have counseling access and receptiveness?
- Do counseling services improve emotional management among older people?
- Do counseling services improve dignity among older people?
- Do counseling services affect decision-making processes among older people in Singapore?

## Methods

### Study Design and Study Area

This study uses a mixed method research design by combining quantitative and qualitative approaches to explore the life experiences of Singaporeans older than 60 years and interpret their lived experiences, such as interactions with qualified counselors accredited by the Singapore Association of Counseling and Singapore Association of Social Workers or social workers. Data are gathered using an exploratory sequential mixed methods design, thereby broadening the scope and dimension of the study. Both qualitative and quantitative approaches offer exceptional strengths that will facilitate robust findings in this study [24-26].

The 5 Singaporean estates with a high number of individuals aged 60 years and older [27] were identified. Bedok and Tampines in the eastern part of Singapore have the largest older populations, with 62,750 and 43,700 people, respectively, followed by Hougang in the northeastern part of Singapore with 41,920 older residents and Ang Mo Kio in the northern part of Singapore with 41,390 older residents. Given the high population of older individuals in Bedok and Tampines, older individuals receiving some form of preventive, primary, or long-term care in the community through voluntary welfare organizations (VWOs) have been recruited for this study. Furthermore, a total of 78 senior activity centers (SAC) in Singapore (a VWO with 10 SACs is located in eastern Singapore) were selected for this study.

### Study Population

The study population comprises individuals aged 65 years and older from various parts of Singapore.

### Inclusion and Exclusion Criteria

Textbox 1 shows the inclusion and exclusion criteria for selecting the study participants.

**Inclusion and exclusion criteria.**

**Inclusion criteria**

- Participants must be 65 years of age or older on the first day of selection.
- Participants must be living in the community.
- Participants must be interested in the study and receive some form of preventive, primary, or long-term care services.
- Participants must be willing to attend counseling sessions in emotional management and decision-making processes.

**Exclusion criteria**

- Older individuals who are currently admitted to a nursing home.
- Older individuals who are mentally incapacitated based on a history of confirmed mental disorder or unable to understand the research protocol when briefed by the researcher.

### Sample Size Calculation and Sampling Technique

The sample size for the quantitative phase was estimated based on the total population of older people in the study locations, the 95% CI, a precision error of 5%, and estimates from previous related studies on the proportions of participants with satisfactory accessibility and receptiveness levels toward counseling [28]. Specifically, the sample size was estimated by using the formula for analytical and cohort studies presented below:


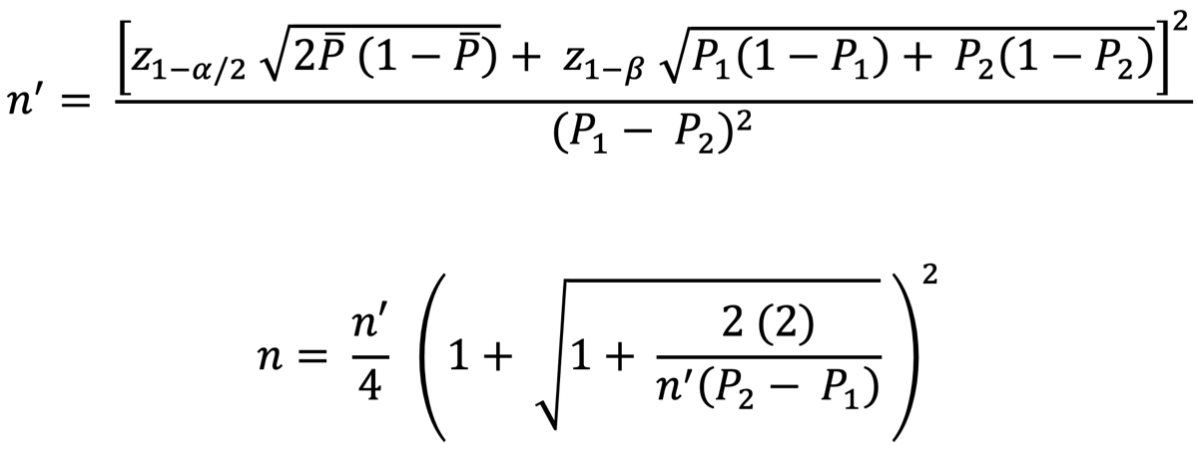


Where

n = sample size

*z*_1–_*_α_*_/2_ = *z* statistic for 95% CI = 1.96

*z*_1-_*_β_* = *z* statistic for 90% power = 1.28

*P*_1_ = Proportion of participants with satisfactory accessibility and receptiveness levels toward counseling in 1 group and

*P*_2_ = Proportion of participants with satisfactory accessibility and receptiveness levels toward counseling in 1 group

*P*_1_ and *P*_2_ were obtained from previous studies reporting the proportions of participants with satisfactory accessibility and receptiveness levels toward counseling [15,28]. The obtained values were substituted into the equation to calculate the required sample size (n). The computed sample size was adjusted for eligibility by assuming that approximately 30% of the participants have access to and are receptive to counseling. Furthermore, a nonresponse rate of 20% was also considered before deciding on the final sample size.

The probability proportionate to size method was used to allocate sample size for each region and part of Singapore to facilitate proportionate distribution of older individuals receiving some form of preventive, primary, or long-term care in the community through VWOs. Thereafter, a simple random sampling technique was used to obtain the required number of participants from each region. Meanwhile, purposive sampling was used in selecting the SACs.

### Qualitative Phase: Study Instrument and Interview Session

A small sample of participants was invited to participate in the qualitative phase, with an equal number of samples from each SAC. As described by previous scholars, 10% of the computed sample size for the quantitative phase was recruited for the interview session. However, an equal number of older individuals were selected from each SAC in various parts of Singapore to participate in the study. The interview session was performed using a semistructured questionnaire. The instrument enables participants to share their views and experiences of counseling services, while some of the responses can be measured numerically.

Six components were explored during the interview session, including the use of the Advanced Care Planning (ACP) booklet among older people, the Trait Meta Mood Scale (TMMS), older people’s accessibility and receptiveness toward counseling, and the improvement of older people’s dignity in emotional management and decision-making. The interview guide was reviewed in subsequent sessions to explore any new emerging themes.

### Quantitative Phase: Instrument Development and Administration

This study uses the ACP and the TMMS instruments. The ACP is a booklet that was designed by the Agency for Integrated Care for recording and planning health and personal care. It comprises 5 main sections that focus on important lifestyle activities, concerns about current health status, views toward life and health, end of life, and preferred end-of-life care. The items are presented as either multichoice options or on a 5-point Likert scale ranging from 1 to 5, depending on the section. For the section on lifestyle activities (4 items), 1=“there is little point in being alive without the quality of life,” while 5=“I would like to be able to live for as long as possible.” For the section on participants’ views toward end of life (4 items), 1=“strongly disagree” and 5=“strongly agree.” On the other hand, the section on preferred end-of-life care is presented as multichoice options with 9 items.

Meanwhile, the TMMS is a 24-item instrument that was developed to measure meta-mood experience [29]. The TMMS is considered suitable for this study as it is the most used self-administered measurement for individuals’ emotional management [29]. The instrument comprises 3 subscales (Attention to Feelings, Clarity of Feelings, and Mood Repair) with 8 items each that evaluate the 3 meta-mood experience components with a 5-point Likert scale ranging from 1=“strongly disagree” to 5=“strongly agree.” The Attention to Feelings Subscale measures attention to personal and external moods and emotions; the Clarity of Feelings Subscale assesses personal abilities to discriminate feelings among one another; and the Mood Repair Subscale evaluates older individuals’ ability to perform self and external regulations [30].

A structured questionnaire was also developed to assess participants’ views regarding counseling and its impact on their dignity and decision-making process. This was based on the themes identified in the qualitative study and an in-depth review of several studies in the literature. Operational definitions were also developed based on the themes obtained. Three experts assessed the questionnaire for content validity. Thereafter, the instrument was distributed to 10 social workers or counselors for face validity assessment. The comments received were reviewed and necessary modifications were made. The final questionnaire was presented in English, and no translation into local languages was conducted. Pretesting was performed on 20 older individuals from selected SACs for internal consistency and reliability. Findings from the experts’ opinions and pilot testing were used to prevent potential ceiling and floor effects from the questionnaire items that may affect accurate data interpretation. The developed questionnaire was distributed to randomly selected older individuals at all selected SACs. The data obtained from the quantitative and qualitative approaches were combined to provide a more robust assessment of the study. Table 1 depicts the research matrix.

**Table 1:** Research matrix.

Research objectives	Quantitative	Qualitative
To assess advance care planning among older people and their perception of life and health, end of life, and end-of-life care	✓	✓
To explore older people’s accessibility and receptiveness toward counseling		✓
To elucidate older people’s perspectives on counseling and its impact on emotional management and decision-making		✓
To assess older people’s competencies in emotional management	✓	
To propose an intervention model for enhancing older people’s well-being and dignity through psychological counseling	✓	✓

### Ethical Considerations

Ethical considerations in this study entail 2 key ethical principles: honesty and respect for human rights [27,31-33]. Certain ethical questions need to be addressed, since older individuals are research participants. The privacy, safety, and confidentiality of participants’ data are guaranteed. Hence, both informed and signed consent were obtained before the interview and questionnaire administration. A cover letter with vital information on the research objectives was attached to the questionnaire and interview guide. This study protocol has been approved by the Universiti Sains Malaysia Human Research Ethics Committee (USM/JEPeM/21110760).

### Management

Participants’ anonymity will be protected in this study [34-36]. Data will be kept confidential to ensure that the identities of older individuals are protected [37-39]. Research instruments and clinical records containing participants’ data will be represented in a coding system. Thus, participants’ private information will not be revealed when submitting the data and findings to regulatory institutions or sponsors [40,41]. Only the researchers will be able to access the participants’ records and the coding system. Two copies of consent forms were provided to be signed by the researcher and each participant. All the files will be stored in a single folder in a secured location.

This research poses no risk to the participants. No personal expenses will be incurred by the participants during the study. Furthermore, no incentives or financial compensation will be given for participation. The research budget will be used for any further expenses. This study is based on voluntary participation and has no implications for the participants’ counseling if they decide not to participate. Older individuals can decide to withdraw from the research regardless of time, if desired, without any consequences to their counseling or care at the SACs. The expected research findings might be published in a journal article or presented at conferences or meetings; however, participants’ identities will remain confidential.

### Quality Control Measures

A number of methodological resources, such as mixed method study design, inclusion criteria, and random sampling, were used in this study to minimize bias and ensure quality data collection. The interview session was performed by the principal researcher, while trained enumerators assisted in the quantitative phase of data collection. The enumerators were trained during the pilot test to familiarize them with the research instrument. To further ensure quality control, the principal researcher assessed the enumerators periodically to see if they were following the survey protocol. If necessary, the enumerators also assisted the participants while they completed the questionnaire to minimize missing data. Quality control was assessed in the retrieved questionnaires based on the quality and completeness of the data.

### Data Analysis

**Figure 1:**
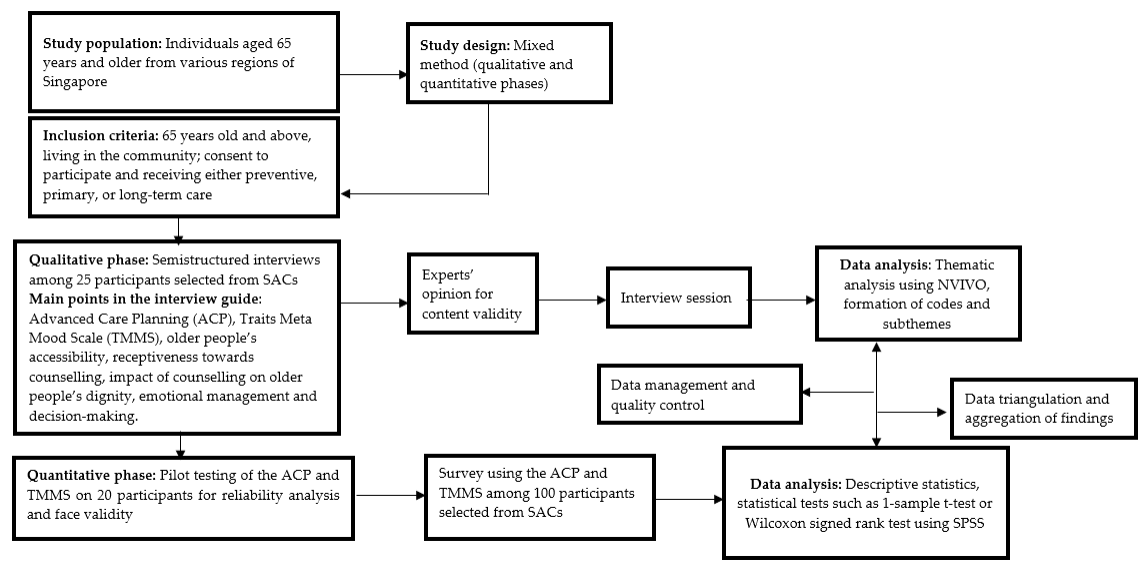
Sequential explanatory approach of the research. ACP: Advanced Care Planning; SAC: senior activity center; TMMS: Trait Meta Mood Scale.

Findings from the qualitative study will be transcribed and coded. Interview transcripts will be analyzed using NVivo (version 12; QSR International). The identification, coding, and categorization of meaningful patterns into themes and subthemes will be performed to facilitate the construction of a structured questionnaire for the quantitative study. For the quantitative phase, the internal consistency of the questionnaire items will be evaluated using a reliability test based on Cronbach α values. Items will be considered acceptable and reliable if the Cronbach α value is equal to or greater than .7. Participants’ background information and other sections in the questionnaire will be analyzed using descriptive statistics. Continuous variables will be subjected to normality tests using the Smirnov-Kolmogorov test and presented as means (SD) or medians (IQR) accordingly. Statistical tests such as a 2-tailed, 1-sample *t* test or Wilcoxon signed rank test will be applied. A *P* value of .05 will be considered statistically significant, and all empirical analyses will be performed using SPSS (version 25; IBM Corp). The flow of the research process, including the vital concepts, is presented in Figure 1.

### Timeline

Older individuals for the quantitative study have been recruited, and the phase was completed in February 2022. Participant recruitment for the quantitative phase commenced immediately upon executing the qualitative phase. The quantitative phase began on March 22, 2022, and was completed in May 2022. Data analysis was performed in September 2022, and the research was completed in March 2023.

## Results

We have obtained ethical approval to conduct this study from the Universiti Sains Malaysia Human Research Ethics Committee (USM/JEPeM/21110760). The qualitative phase, which involved a semistructured interview, was conducted from January 14, 2022, to February 16, 2022, among 25 individuals aged 66-86 years. Thematic analysis of the data is still ongoing. Meanwhile, the quantitative phase commenced on March 22, 2022, with 100 participants providing signed and informed consent to participate in the study. The face-to-face administration of the ACP and TMMS instruments and data collection closed on May 10, 2022. Data analysis and manuscript writing was performed in March 2023.

## Discussion

### Expected Findings

Old age comes with various losses of functions and roles, thereby leading to an increased reliance or dependency on others. Thus, the first objective of this study is to use mixed methodology in assessing older people’s perspectives on ACP, life and health, end of life, and end-of-life care. The ACP instrument is a booklet designed by the Agency for Integrated Care for recording and planning health and personal care, and the main sections comprise important lifestyle activities, concerns about current health status, views toward life and health, end of life, and preferred end-of-life care. Findings from this study will elucidate participants’ views of these pertinent aspects of ACP.

Policymakers in Singapore have recently highlighted the need to provide a conducive environment for older people to age effectively and to be independent for as long as possible [20]. For instance, it is estimated that by 2030, due to the nation’s ultralow fertility rate, those older than 65 years will be supported by only 2.1 million Singaporeans of working age [27]. Hence, the results will assist to understand if participants’ views align with the government’s position to ensure improved independence among the nation’s aging population.

In addition, information about perceived social support, the connection between health and exercise, and current health status or comorbidity will be gleaned from the ACP instrument. Perceived social support is an important predictor of quality of life and satisfaction with life among adults aged 65 years and older [42]. Understanding how older people define physical activity has also been associated with the perceived benefits of healthy aging [43,44]. In terms of health status, a recent study on the epidemiologic characteristics of multimorbidity in Singapore’s aging population found that hypertension and lipid disorders were among the most prevalent disease combinations [45]. Singapore was also reported to have a higher prevalence of multimorbidity compared to several countries worldwide [46], with more than 19% of the population having multimorbidity. Thus, participants’ concerns regarding their health status and present comorbidity will add to the current knowledge pool.

The second objective focuses on qualitative analyses of participants’ perspectives on counseling, awareness and receptiveness toward counseling services, and potential barriers to seeking counseling assistance. Counseling and psychotherapy are confidential conversations between a counselor and a client that are performed to provide solutions to problems [47,48]. Factors such as perception and attitude toward counseling, affordability, financial status, and accessibility are some of the consistent factors influencing receptiveness toward counseling among older individuals [49-51]. Given the limited information on the aforementioned aspects in the Singaporean context, findings from this study will provide opportunities for relevant bodies to ascertain the factors to focus on in enhancing the receptiveness toward counseling among older people.

Our third objective was to understand older people’s perspectives on dignity and how their emotional management and decision-making are influenced by counseling. End-of-life patients’ dignity is a vital theme in the context of palliative care, and health care professionals are expected to address complicated decisions on specific aspects of patients with terminal illnesses [52,53]. Given that older people tend to be more vulnerable to loss of dignity following advanced age and deteriorating health, dignified care is a significant health care goal that ought to be preserved during counseling interventions, especially for older people [54]. Previous studies conducted elsewhere found that terms such as *self-worth*, *self-recognition*, and *self-expression* were used by older participants in describing dignity [55,56]. We expect the participants in this study to use similar terms but tailored to the nation’s context given the different cultural backgrounds compared to Western perspectives.

In terms of the impact of counseling on emotional management and decision-making, diverse views ranging from positive to negative effects may be conveyed by participants since they might have faced challenges in seeking counseling assistance. For instance, a previous study conducted in Tehran, Iran, found that psychological counseling on mental well-being had a positive impact on emotion and self-management ability among older women [57]. Dai et al [58] also performed a meta-analysis of studies conducted among Chinese populations and found that psychologically based therapies were effective in ameliorating depression symptoms among older people. The contents of the ACP instrument used in this study entailed pertinent topics on mental health, the selection of treatment goals (comfort-focused vs full treatment), available care options in the community, and available care options during end of life, which might have a positive effect on emotional management and decision-making. However, difficulties in assessing counseling services or challenges are faced during the counseling process.

The fourth objective of this study emphasized participants’ competencies in emotional management after counseling assistance. This objective will entail both a semistructured interview and a quantitative analysis by using the TMMS, which focuses on participants’ attention (perceived attention paid to personal emotional conditions), clarity (perceived understanding of personal emotional conditions), and repair (perceived ability to regulate personal emotional conditions). The TMMS is one of the most commonly used self-administered measurements for perceived emotional competence in older people [29]. It provides the opportunity to bridge current empirical gaps on emotions and to elucidate developmental trajectories throughout a person’s life span. Based on the alignment between the ACP contents and items in the TMMS, as well as findings from previous local studies on the impact of counseling [20], we expect older people’s competencies in emotional management and decision-making to improve after seeking counseling assistance. Results from this study may assist policymakers in deploying strategies to promote mood repair and emotional management in the Singaporean aging population to improve their receptiveness toward counseling interventions.

Overall, this study takes a holistic look into novel approaches to developing an effective counseling intervention for older people in Singapore. By considering older people’s perspectives on pertinent issues regarding dignified care, life and health, end-of-life care, and the impacts of counseling, the challenges and prospects of available counseling services offered at various centers in Singapore can be elucidated. Moreover, patients, health care professionals, and social workers play pivotal roles in decision-making processes and ensuring optimum care during routine hospitalization of older patients’ at their end of life. We anticipate that the data gleaned from this study will provide important insights regarding the approaches to developing a feasible and acceptable counseling intervention for older individuals in Singapore, as well as incorporating the intervention into routine care and accentuating potential barriers to seeking counseling assistance among this older population.

### Implications

The findings in this study, particularly older people’s perspectives on ACP, dignity, life and health, end of life, accessibility and receptiveness toward counseling, and competencies in emotional management and decision-making, should be beneficial to relevant policymakers in Singapore. Counseling is still an emerging field in Singapore, and there is a lack of mandatory counseling service regulations in Singapore. Given the various counseling services provided by professional organizations and private individuals, policymakers may consider the findings in standardizing the service training and counseling content for the increasing older population in Singapore.

This research is also promising in enhancing the target group’s health, cognitive, and social functioning aspects. Given the current aim of the Singaporean government to tailor counseling for older people toward their cultural backgrounds [20] and coupled with the fact that older Singaporeans have less access to Western perspectives [59], the findings may assist in incorporating their perspectives into developing effective counseling interventions. Diverse perspectives have been reported to affect older individuals’ receptiveness to counseling interventions [55,57].

Another important implication of this study is for the social workers, counselors, and health care professionals who are expected to deliver effective services to older individuals. The findings on older people’s perspectives on ACP, dignity, life and health, and end-of-life care could be used to bridge the gap between the various players toward holistic patient-centered care. Moreover, counselors are expected to upgrade their professional knowledge and skills to ensure that older people live with dignity and access essential health and social services for optimal autonomy and independence. Thus, counselors in Singapore may find this study an opportunity to equip themselves with clinical skills to counsel older people.

### Strengths and Limitations

Given the paucity of research on counseling as an intervention to enhance older people’s dignity within the community, this study has attempted to fill in the research gap between counseling and Singaporean government policy on aging in place, thus adding to the current body of knowledge and literature in the research area both at local and global levels. The variables and specific events investigated in this study, such as receptiveness and accessibility to counseling centers, improved emotional management and decision-making, and quantitative assessment of perceived attention, clarity, and mood repair among older individuals, represent the novelty of this research. To date, only a few studies have considered the lived experiences and interactions of older adults with counselors or social workers. This study represents the first attempt to use mixed research methods to bridge the research gap. This combined approach will facilitate the collection of vital data through in-depth interviews and survey methods to elucidate older individuals’ lived experiences and interactions with qualified and Singapore Association of Counseling and Singapore Association of Social Workers–accredited counselors or social workers.

However, the study design enabled the recruitment of participants with similar characteristics, and the inclusion and exclusion criteria used in selecting the study participants might have excluded older individuals with pertinent information on the research topics. Furthermore, the use of semistructured interview sessions also has its limitations, as respondents might be unwilling to provide in-depth information to the questions being asked. Moreover, the ACP instrument used in the qualitative phase has some closed-ended questions in which participants were not free to express their opinions. This might contribute to a degree of response bias. Last, this study has a cross-sectional design, and the findings will only reveal the association between the studied variables, such as accessibility and receptiveness toward counseling, perspectives on dignity and barriers to counseling assistance, and the related impact on emotional management and decision-making. In other words, no causal relationships can be inferred. Longitudinal, prospective, and randomized control study designs are more likely to generate the required data to infer causal effects and relationships between the studied concepts.

### Conclusion

Accumulated evidence reflects the paucity of data on the impact of counseling on older people’s dignity and emotional management in Singapore. Moreover, their accessibility and receptiveness toward counseling remain underreported. With the rising older population and the need for better care and effective aging, this research will be pertinent in elucidating the role of counseling in addressing the aforementioned issues. Policymakers and relevant bodies can use the findings to implement a model that will enhance older people’s receptiveness toward counseling and derive vital benefits from such an intervention.

## Abbreviations

ACP: Advanced Care Planning
SAC: senior activity centers
TMMS: Trait Meta Mood Scale
VWO: voluntary welfare organization

## Data Availability

All data generated or analyzed during this study will be included in the published article and its supplementary information files.
